# Supplementary figures and images for: Major histocompatibility complex class I evolution in songbirds: universal primers, rapid evolution and base compositional shifts in exon 3
Source: PeerJ. 2013 Jun 11;1:e86. doi: 10.7717/peerj.86 (PMC3685324; doi:10.7717/peerj.86)

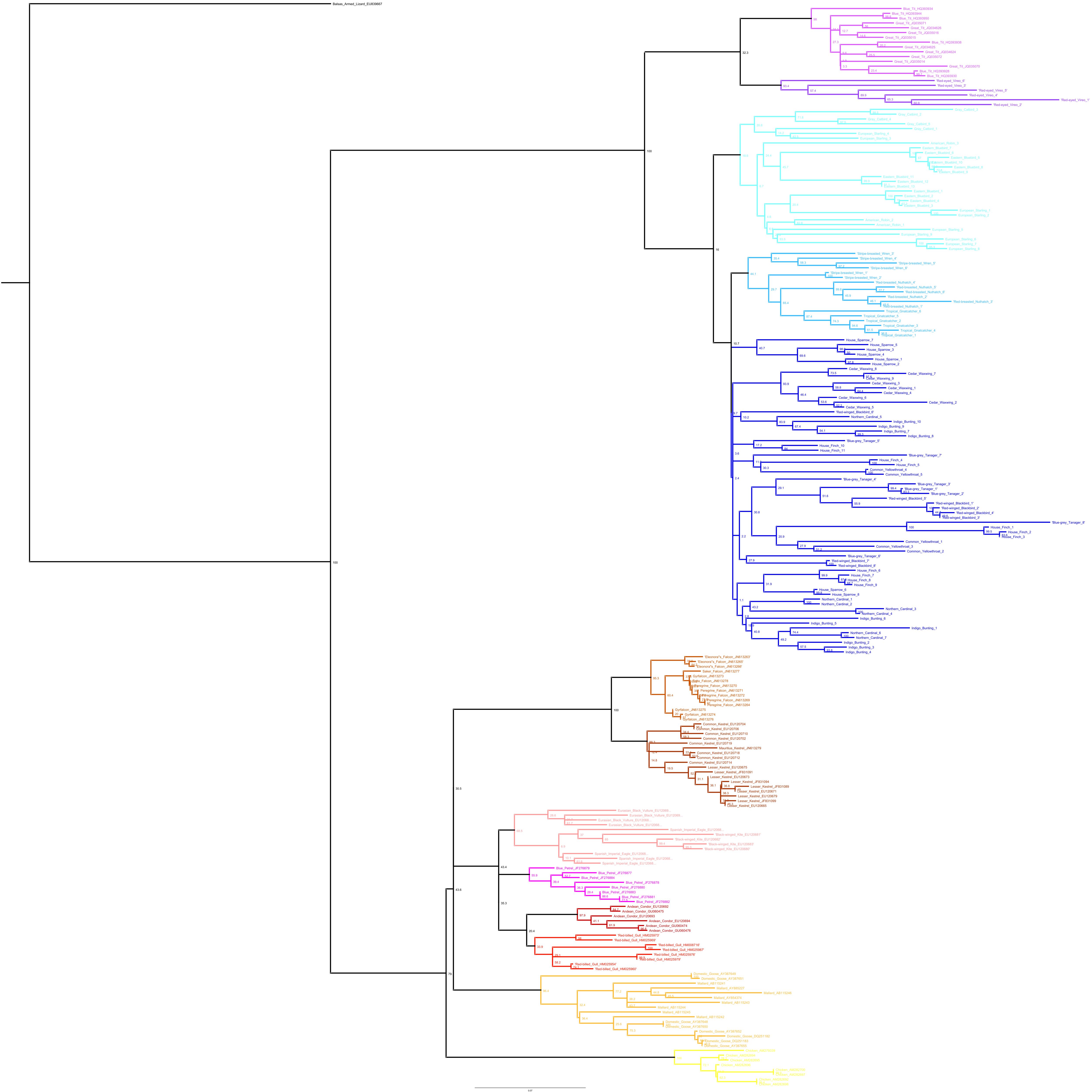

Supplement: Figure S1 [file peerj-01-86-s001.jpg]
